# Investigating the association of testosterone with survival in men and women using a Mendelian randomization study in the UK Biobank

**DOI:** 10.1038/s41598-021-93360-z

**Published:** 2021-07-07

**Authors:** C. M. Schooling, J. V. Zhao

**Affiliations:** 1 School of Public Health, Li Ka Shing, Faculty of Medicine, The University of Hong Kong, 1/F, Patrick Manson Building (North Wing), 7 Sassoon Road, Pokfulam, Hong Kong, China; 2 Graduate School of Public Health and Health Policy, City University of New York, New York, NY USA

**Keywords:** Epidemiology, Cardiovascular diseases

## Abstract

Life expectancy in the developed West is currently stagnated and remains shorter in men than women. Well-established evolutionary biology theory suggests lifespan trades-off against reproductive success, possibly sex-specifically. We examined whether a key driver of reproductive success, testosterone, affected survival using a Mendelian randomization longevity study in the UK Biobank to obtain unbiased estimates, along with control exposures. We applied published genetic instruments for testosterone to obtain inverse variance weighted estimates of associations with survival to (i.e., age at) recruitment, in 167,020 men and 194,174 women. We similarly obtained estimates for a positive control (smoking initiation), and a negative control (absorbate), a marker of vitamin C metabolism. Testosterone was associated with poorer survival (0.10 years younger at recruitment per effect size of testosterone, 95% confidence interval (CI) 0.004 to 0.20). As expected, smoking initiation was also associated with poorer survival (0.37 years younger, 95% CI 0.25 to 0.50), but not absorbate (0.01 years younger, 95% CI − 0.09 to 0.11). Several aspects of a healthy lifestyle (low animal fat diet) and several widely used medications (statins, metformin, dexamethasone and possibly aspirin) may modulate testosterone. Explicitly designing interventions sex-specifically based on these insights might help address stagnating life expectancy and sexual disparities.

## Introduction

Sexual dimorphism in lifespan is widely evident, including among humans, contemporaneously and historically^1,2^. Many reasons have been advanced for shorter life expectancy in men than women, from gendered health and health care seeking behavior^3^ through sex-specific nutrient metabolism and steroid hormones^4^ to sex-specific sexual selection pressures^5,6^, but few of these have been explicitly exploited as a means of promoting healthy aging in both sexes. As the long-term trend of increasing life expectancy in the West now appears to be stagnating^7^, reconsidering overlooked targets and their implications for the design of interventions might bear consideration.

An obvious modifiable difference between men and women is levels of sex hormones. Estrogen has been intensively investigated as a protective factor that might explain differences in life expectancy by sex. Observational evidence concerning benefits of exogenous estrogen in women was very promising^8^. Large-scale trials of estrogen in women and men were stopped early for harm or lack of benefit^9^. Discrepant findings in trials compared to observational studies have been ascribed to overlooked confounding by socio-economic position in the observational studies^9^. Promising observational evidence exists for survival benefits of endogenous testosterone in women^10–12^ and men^13,14^, although some inconsistencies exist^15–17^. Testosterone in men falls with aging and ill-health^18–20^, likely generating residual confounding, as health status is difficult to adjust for comprehensively. A trial of the cardiovascular safety of exogenous testosterone in women has been successfully conducted^21^. A small trial of exogenous testosterone in frail older men was stopped early because of adverse events in the testosterone arm^22^, but is difficult to interpret. A larger trial of exogenous testosterone on cardiovascular disease is now underway in men^23^. Theoretically, the expectation for effects of testosterone on health might be in a similar direction to those found for estrogen because of the well-established Darwinian evolutionary biology trade-off between reproduction, and its drivers, on the one hand, and survival, on the other hand^24–26^.

Here we conducted a Mendelian randomization (MR) study of the effects of testosterone on survival in men and women. MR studies take advantage of the random allocation of genetic material at conception to obtain less confounded estimates^27^. Specifically, MR studies use genetic variants as proxies of the exposure, and compare outcomes according to genetically predicted differences in exposure, i.e., use an instrumental variable design with genetic instruments. We used existing genetic predictors of testosterone and the control exposures^28–30^ and applied them to a study of longevity. Longevity studies compare characteristics of survivors to older ages with characteristics of younger people because lifetime harmful factors inevitably become less common with increasing age^31^. In contrast, observational prospective cohort studies comparing mortality incidence after recruitment are open to selection bias from any deaths prior to recruitment having already depleted the susceptibles^32^ which may attenuate estimates even to the extent of suggesting no benefit of proven healthy interventions^33^. As such, longevity studies, take advantage of the changing structure of risk factors with age to obtain estimates of effects on mortality free from selection bias^34^. In addition, as this is a hypothesis driven study, we also included positive and negative control exposures. Smoking initiation was used as a positive control outcome because smoking is very well-known to be addictive and to substantially reduce survival to old age^35^. A marker of vitamin C metabolism, absorbate, was used as a negative control outcome because vitamin C is no longer thought to affect health^36^.

## Results

In total 167,020 men and 194,174 women from the UK Biobank were included. Almost all the genetic variants used as instruments for the exposures (testosterone (125 variants in men and 254 variants in women), smoking initiation (361) and absorbate (1)) were available for the outcome. Two variants for smoking initiation were replaced by highly correlated (r^2^ > 0.9) variants (rs2587507 replaced by rs745571, and rs2359180 by rs768576) obtained from LDlink (https://ldlink.nci.nih.gov/). The average F-statistic for testosterone was 128.6 in men and 83.6 in women, and for smoking initiation was 44.9. As expected, smoking initiation was associated with younger age at recruitment (Table 1), corresponding to poorer survival. Similarly, as expected absorbate was unrelated to age at recruitment (Table 1). Overall, testosterone was associated with younger age at recruitment, i.e., poorer survival (Table 1), with slightly larger estimates in men than women (Table S1). Estimates were similar in sensitivity analysis using age at recruitment as a categorical variable (age 60 years or less) (Table S2) and using sensitivity analysis (Table S1).

**Table 1:** Differences in age at recruitment to the UK Biobank for published genetic predictors of testosterone^28^, smoking^29^, and vitamin C (absorbate)^30^ using Mendelian Randomization inverse variance weighted estimates.

Exposure type	Exposure	Difference in age at recruitment (years)	95% CI	*p* value
Positive control	Smoking initiation compared to not	− 0.37	− 0.50 to − 0.25	5.2e−9
Negative control	Absorbate (rs33972313)	− 0.01	− 0.11 to 0.09	0.79
Exposure	Testosterone (standard deviation)	− 0.10	− 0.20 to − 0.004	0.04

## Discussion

Despite observational studies suggesting that testosterone might promote longer life^10–14^, our study with greater robustness to confounding and selection bias, consistent with theoretical expectations from evolutionary biology^24,25^, suggests that testosterone reduces survival. Our study also showed smoking initiation reduced survival as would be expected^35^.

Our findings differ from previous observational studies which suggest that endogenous testosterone might improve health and lifespan^10–14^. However, these studies are open to both confounding by health status^18,19^ and selection bias from inevitably only recruiting survivors^32^. Our findings are more consistent with previous MR studies suggesting exogenous testosterone increases cardiovascular disease^37,38^, and corresponding warnings from regulators (https://www.fda.gov/drugs/drug-safety-and-availability/fda-drug-safety-communication-fda-cautions-about-using-testosterone-products-low-testosterone-due). Our findings are also consistent with the observation that in one of the few primate species with no age or sex difference in testosterone levels, there is also no sex difference in age-dependant survival.

Several potential mechanisms could underlie our findings. Experimental studies suggest testosterone increases coronary plaque volume^39^ and coagulation^40^ while impairing endothelial function^41–43^. Testosterone increases vulnerability to hormone related cancers, including prostate cancer^28^, breast cancer^28^ and endometrial cancer^28^. Testosterone is also increasingly acknowledged to be an immune-suppressant^44^, potentially increasing susceptibility to cancer^45^, as well as to infectious diseases^44^, such as coronavirus disease 19. Testosterone may also induce impulsive behaviour^46^.

### Relevance to interventions

Given the importance of testosterone to reproductive success, testosterone is likely responsive to environmental indicators of suitable conditions for procreation, and hence a modifiable target of intervention. Serendipitously, many aspects of a healthy lifestyle and several widely used interventions reduce testosterone. A healthy diet, i.e., low-fat, high fiber, high soy, and low animal fat reduces testosterone in men^47–49^ and women^50^. Exercise and weight loss reduce testosterone in women^51–53^, but possibly less so in men^54,55^, although severe caloric restriction (50% energy requirements) reduces testosterone in men^56^. One of the most effective medications for preventing and treating cardiovascular disease, i.e., statins, reduces testosterone^57^. The first-line treatment for diabetes, metformin, may reduce testosterone in women^58^, but not men^59^. Aspirin may also reduce testosterone^60^. Another essential medicine, dexamethasone reduces testosterone^61,62^. As such, many current interventions to promote healthy aging do happen to reduce testosterone, particularly in women. Explicitly, searching sex-specifically for interventions based on this insight might facilitate the search for new means of promoting healthy aging by identifying at an early stage any potential interventions that are likely to be unsuccessful, and where interventions should be sex-specific.

### Limitations

Although this study used an innovative approach to generate an unbiased assessment of the effect of testosterone on survival, it has several limitations. First, MR has stringent assumptions including that the genetic instruments predict the exposures, hence our use of published instruments^28–30^, that the instruments are independent of exposure outcome confounders, which stems from the use of genetic instruments not related to confounders such as health status, lifestyle and socioeconomic position, and that the instruments only affect the outcome via the exposure, hence our use of sensitivity analysis and control exposures. Second, we assumed a linear relation of survival with age when it is exponential. However, at the relatively young age (mean age 57 years, intended recruitment age 40 to 69 years) considered here such an approximation is less of an issue than in old age. A sensitivity analysis using survival to age 60 years as the outcome gave a similar interpretation for genetically predicted testosterone (Table S2). Third, the samples used for exposures and outcome overlap. However, that would be expected to bias towards the null^63^, so our estimates of associations may be conservative. Moreover, the potential bias is less of concern as we did not use weak instruments to predict exposures^63^. Fourth, our study gives lifetime effects of endogenous testosterone up to age ~ 57 years rather than the effect of an intervention. However, the estimates for testosterone can be contextualized by the estimates for smoking. Fifth, longevity studies have been criticized for not taking account of cohort effects^34^. However, there is no reason to think that genetics are susceptible to cohort effects in the UK Biobank. Sixth, canalization, i.e., compensation for genetic effects might exist. However, this would likely bias towards the null. Seventh, the UK Biobank is not a population representative study. However, we used a study of longevity rather than a traditional prospective cohort study to avoid selection bias. Eighth, the relatively young age at recruitment, and correspondingly high survival means this study lacks power, so we focused on major determinants of healthy aging, but were not able to ascertain if the effects of testosterone were greater in men than women. Ninth, we were unable to replicate this study using a genome wide association study of life span or parental life span. Such studies typically do not include the X chromosome, which is important for testosterone. They also typically recruit in middle-age, i.e., recruit survivors, instead of taking account of those who have already died before recruitment, so are open to selection bias. Tenth, the effects of testosterone may vary by age. However, causes are generally expected to act consistently, although they may not be relevant in all situations^64^. Testosterone in men falls with age^20^, so these findings could be relatively less relevant to older men, but absolute risk of death increases with age.

## Conclusions

Consistent with well-established evolutionary biology theory^24–26^ testosterone appeared to reduce survival. Serendipitously, many existing strategies for promoting healthy aging reduce testosterone, particularly in women. Explicitly using an established theory as a guide might facilitate the search for new interventions, and draws attention to the importance of sex-specific interventions.

## Methods

### Data sources

The UK Biobank recruited half a million people intended to be aged 40 to 69 years from across Great Britain in 2006–10^65^. Average age is about 57 years and just over half the participants are women. We extracted overall and sex-specific genetic associations with age at recruitment from publicly available UK Biobank summary statistics in white British (http://www.nealelab.is/uk-biobank). These specific summary statistics were generated from linear regression adjusted for sex and the first 20 principal components, after excluding poor quality samples. Where relevant genetic information was not available from these summary statistics, we similarly generated corresponding information from the UK Biobank individual data.

### Exposures

We used published sex-specific genetic predictors of testosterone (125 variants for men and 254 for women)^28^. We used published genetic predictors of smoking initiation (361 variants)^29^, and of vitamin C metabolism (1 variant)^30^.

### Outcome

In this study, we used age at recruitment to the UK Biobank as a proxy for survival, as there is no particular reason, apart from prior death (i.e., lack of survival), why genetics should generate differences in willingness to participate by age. So, harmful exposures would be expected to be associated with younger age at recruitment.

### Statistical analysis

We obtained the F-statistics for the genetic instruments using an established approximation (square of genetic variant on exposure divided by its variance)^66^. As an F-statistic of 10 or less is usually taken as indicating a weak instrument. We aligned genetic associations on the same allele for exposures and outcome. We included palindromic and non-biallelic genetic variants because the genetic associations with exposures and outcome were obtained in the same study or have been used in the same study. We used proxies, as necessary. We meta-analyzed genetic variant specific Wald estimates (ratio of genetic variant on outcome to genetic variant on exposure) using inverse variance weighting (IVW) with multiplicative random effects. IVW estimates assume balance pleiotropy^67^. We used estimates with different assumptions as sensitivity analysis. The weighted median has a majority valid assumption and assumes > 50% of the genetic variants are valid instruments, while the contamination mixture method has a contrasting plurality valid assumption^67^. MR-Egger detects pleiotropic effects acting on the outcome other than via the exposure, but assumes that the genetic instruments do not act via confounders of exposure on outcome^66^. To avoid pleiotropic effects of sex hormone binding globulin, we used established genetic predictors of bio-available testosterone in men and total testosterone in women, as previously^68^. Bio-available testosterone may also be a more sensitive indicator that total testosterone of active testosterone in men^69^. Where necessary we combined estimates for men and women using IVW meta-analysis.

Given this is a hypothesis driven study, with a positive and a negative control, we used a statistical significance level of 0.05. All statistical analysis was conducted using R version 3.6.1 (The R Foundation for Statistical Computing, Vienna, Austria). The MendelianRandomization R package was used for the MR estimates and the metafor R package for meta-analysis. Estimates of genetic associations were taken from publicly available UK Biobank summary statistics, except for the unavailable associations which were based on individual level genetic associations from the UK Biobank obtained under application #42468. All UK Biobank data was collected with fully informed consent.

## Supplementary Information

Supplementary Tables.

## Data Availability

JVZ and CMS had full access to the data and take full responsibility for the results.
