# Supplementary material for: Investigating the association of testosterone with survival in men and women using a Mendelian randomization study in the UK Biobank
Source: Sci Rep. 2021 Jul 7;11:14039. doi: 10.1038/s41598-021-93360-z (PMC8263740; doi:10.1038/s41598-021-93360-z)
Supplement: Supplementary file 1 — Supplementary Tables. [file 41598_2021_93360_MOESM1_ESM.docx]

**Title:** Testosterone and survival in men and women: A Mendelian randomization study in the UK Biobank

**Authors:** Schooling CM*, Zhao JV

**Affiliations:** CM Schooling, School of Public Health, Li Ka Shing Faculty of Medicine, The University of Hong Kong, Hong Kong, China, City University of New York, Graduate School of Public Health and Health Policy, New York, NY, USA

JV Zhao, School of Public Health, Li Ka Shing Faculty of Medicine, The University of Hong Kong, Hong Kong, China

**Contact information** for corresponding author: CM Schooling, PhD.

1/F, Patrick Manson Building (North Wing), 7 Sassoon Road, Pokfulam, Hong Kong

Tel: 852 3917 6732 Email: [cms1@hku.hk](mailto:cms1@hku.hk)

**Supplementary Table 1: Differences in age at recruitment to the UK Biobank for published genetic predictors of testosterone[1] and smoking**[2] **using additional Mendelian Randomization analyses**

| Exposure | Method | Subgroup | Years younger at recruitment | 95% CI | P-value | Intercept p | Q (p-value) |
| --- | --- | --- | --- | --- | --- | --- | --- |
| Smoking | WM | all | -0.35 | -0.52 to -0.17 | 0.0001 |  |  |
| (361 variants) | MR Egger | all | -0.32 | -0.84 to 0.21 | 0.24 | 0.83 | 434.4 (0.004) |
|  | Conmix | all | -0.61 | -0.94 to -0.34 | 0.0003 |  |  |
|  |  |  |  |  |  |  |  |
| Testosterone | IVW | Men | -0.12 | -0.29 to 0.05 | 0.16 |  |  |
| (125 variants for men, 254 variants for women) |  | Women | -0.09 | -0.22 to 0.03 | 0.14 |  |  |
|  | WM | Men | -0.25 | -0.54 to 0.04 | 0.09 |  |  |
|  |  | Women | -0.28 | -0.51 to -0.05 | 0.02 |  |  |
|  |  | all | -0.27 | -0.45 to -0.09 | 0.003 |  |  |
|  | MR-Egger | Men | -0.20 | -0.48 to 0.07 | 0.15 | 0.47 | 154.2 (0.03) |
|  |  | Women | -0.13 | -0.35 to 0.10 | 0.27 | 0.72 | 262.1 (0.32) |
|  |  | All | -0.16 | -0.33 to 0.02 | 0.08 |  |  |
|  | Conmix | Men | -0.34 | -0.59 to -0.01 | 0.049 |  |  |
|  |  | Women | -0.12 | -0.28 to 0.05 | 0.16 |  |  |
|  |  | All | -0.17 | -0.32 to -0.02 | 0.03 |  |  |

WM: weighted median, IVW: inverse variance weighted, Conmix: contamination mixture model

**Supplementary Table 2: Recruitment to the UK Biobank at age 60+ years compared to younger than or equal to 60 years for genetically predicted testosterone[1] using Mendelian Randomization inverse variance weighting estimates**

|  | Odds ratio | 95% CI | p-value |
| --- | --- | --- | --- |
| All | 0.98 | 0.95 to 0.99 | 0.049 |
| Men | 0.97 | 0.93 to 1.01 | 0.13 |
| Women | 0.98 | 0.95 to 1.01 | 0.20 |

1. Ruth KS, Day FR, Tyrrell J, Thompson DJ, Wood AR, Mahajan A, Beaumont RN, Wittemans L, Martin S, Busch AS, Erzurumluoglu AM, Hollis B, O'Mara TA, et al. Using human genetics to understand the disease impacts of testosterone in men and women. Nat Med. 2020; 26(2):252-258.

2. Larsson SC, Mason AM, Bäck M, Klarin D, Damrauer SM, Michaëlsson K and Burgess S. Genetic predisposition to smoking in relation to 14 cardiovascular diseases. European heart journal. 2020.
